# Visualisation of xanthan conformation by atomic force microscopy

**DOI:** 10.1016/j.carbpol.2016.04.078

**Published:** 2016-09-05

**Authors:** Jonathan Moffat, Victor J. Morris, Saphwan Al-Assaf, A. Patrick Gunning

**Affiliations:** a Asylum Research an Oxford Instruments Company, Halifax Rd., High Wycombe, Buckinghamshire, HP12 3SE, UK; b Institute of Food Research, Norwich Research Park, Norwich, NR4 7UA, UK; c Hydrocolloids Research Centre, Institute of Food Science & Innovation, Faculty of Science & Engineering, University of Chester, Parkgate Road, Chester CH1 4BJ, UK

**Keywords:** Atomic force microscopy, Xanthan, Structural conformation, Counterions

## Abstract

Direct visual evidence obtained by atomic force microscopy demonstrates that when xanthan is adsorbed from aqueous solution onto the heterogeneously charged substrate mica, its helical conformation is distorted. Following adsorption it requires annealing for several hours to restore its ordered helical state. Once the helix state reforms, the AFM images obtained showed clear resolution of the periodicity with a value of 4.7 nm consistent with the previously predicted models. In addition, the images also reveal evidence that the helix is formed by a double strand, a clarification of an ambiguity of the xanthan ultrastructure that has been outstanding for many years.

## Highlights

• New AFM imaging methodology BlueDrive™ enabling resolution of xanthan’s helix.
• Visual evidence of the structural composition of xanthan’s helices.
• Confirmation of the effect of counterion screening on structural ordering.

## 1 Introduction

Xanthan is a bacterial polysaccharide produced by *Xanthamonas campestris* (Garcia-Ochoa, Santos, Casas, & Gomez, 2000). The polysaccharide consists of a linear β(1,4) linked d glucose cellulosic backbone substituted with a regular trisaccharide sidechain, containing two mannose (Man) and a glucuronic acid (GlcA), attached on every other glucose at C-3. The charged sidechain consists of βDMan(1-4)βDGlcA(1-2)αDMan(1-. The terminal mannose units may contain a pyruvic acid substitute and the α-linked mannose units may have an acetyl group at position O-6 (Phillips & Williams, 2009). Another two recent studies have shown that there can be more heterogeneity of xanthan’s repeat unit than was previously assumed in terms of the ratio of the charge groups within xanthan’s sidechains depending upon the fermentation conditions: There are 6 different patterns of attachment of pyruvate and acetate groups to the pentasaccharide repeat unit, and the relative abundance of these affects the stability of the ordered structure (Kool, Gruppen, Sworn, & Schols, 2013; Kool, Gruppen, Sworn, & Schols, 2014). It is not clear whether this heterogeneity arises due to intra- or inter-molecular substitution. The charged groups on the sidechains play a vital role in xanthan’s aqueous solubility and also its structural conformation (Phillips & Williams, 2009). In the presence of stabilising counterions, which shield the intramolecular charge–charge interactions, the sidechains fold down compactly against the backbone leading to the formation of a 5-fold ordered helical structure (Norton, Goodall, Frangou, Morris, & Rees, 1984). The ordered structure is much stiffer than the disordered ‘random coil’ conformation. In the helical state xanthan has a persistence length in excess of 100 nm, ranking it amongst the stiffest known biopolymers. Previous studies have proven that electrostatic interactions between the charged groups within xanthan and screening counterions determine its ultrastructural conformation in solution (Matsuda, Biyajima, & Sato, 2009; Bejenariu, Popa, Picton, & Le Cerf, 2010; Brunchi, Morariu, & Bercea, 2014).

Traditional methods have been used widely to investigate the molecular conformation of polysaccharides. Optical rotation, circular dichroism, differential scanning calorimetry and rheology are convenient methods for following the course of disorder–order and order–disorder transitions in response to external variables (temperature, ionic strength, concentration of specific cations and denaturants). X-ray fibre diffraction remains the only technique capable of characterising ordered structures at atomic resolution, provided that the chains are well enough oriented and aligned, but atomic resolution has not yet been achieved for xanthan.

The principle question addressed by the present study is that there has been considerable ambiguity for many years on the detail of xanthan’s secondary structure (Morris, 1998). The initial interpretation of X-ray fibre diffraction data was that it formed a single helix (Moorhouse, Walkinshaw, & Arnott, 1977). A subsequent study (Okuyama et al., 1980), carried out in response to the “two strands = double helix” lobby, examined possible double-helix models. It was concluded that, on the basis of the X-ray evidence alone, it was not possible to assign a double-helix or single helical model for xanthan.

In terms of physical chemical studies the salt-induced disorder–order transition followed first order rather than second order kinetics, which suggested a single helix (Norton et al., 1984). Many groups (Morris, 1998) have equated observed dimerization of xanthan with double-helix formation; but that is not evidence based and it is potentially an oversimplified interpretation. The ambiguity with such methods is the fact that they are *ensemble* measurements. This means that the analytical conclusion is controlled by the ratio of ordered to disordered states, so that they lack a certain degree of sensitivity compared to microscopical techniques, such as atomic force microscopy (AFM). AFM is capable of visualising the structure of individual molecules. The main objective of this study is to provide direct evidence on the nature of xanthan’s secondary structure. The unique advantage of AFM is its ability to visualise directly the topology of polymer networks under near-native conditions, which can be a very powerful complimentary technique to combine with *ensemble* methodologies. An integral study using various biophysical techniques, namely, AFM, gel permeation chromatography with multi-angle light scattering (GPC-MALLS) and intrinsic viscosity measurements by capillary viscometry on the conformation of xanthan, following various different treatments (heating, autoclaving, irradiation and high pressure homogenisation), was recently reported (Gulrez, Al-Assaf, Fang, Phillips, & Gunning, 2012). Several polymer parameters derived from these techniques, such as the radius of gyration (Rg), M_w_, polydispersity, molar mass per unit contour length of the rod (M_L_) and Huggins constant (K_H_) were correlated well with the results obtained by AFM. It was possible to correlate the height measurements obtained by AFM with values close to 1000 Dalton per nanometre (Da nm^−1^) and 2000 (Da nm^−1^) assigned for single and double helix, respectively in agreement with previous reports which solely relied on light scattering measurements (Sato, Kojima, Norisuye, & Fujita, 1984; Sato, Norisuye, & Fujita, 1984). Furthermore, using positively-charged mica (coated with poly-l-lysine) a single strand molecule was trapped in a ‘random coil’ conformation (Gulrez et al., 2012). This is consistent with the widely agreed view that xanthan at low concentration and negligible ionic strength adopts ‘random coil’ conformation.

The present study reveals new images of xanthan at sub-molecular resolution revealing the fine detail of its secondary structure development, which has enabled the process of charge screening to be investigated in a manner never previously reported.

## 2 Experimental

### 2.1 Atomic force microscopy

The atomic force microscope (Cypher AFM, Asylum Research Inc, an Oxford Instruments company, Santa Barbara, CA, USA) was operated in AC mode in aqueous buffers containing different counterions. Buffer 1: 10 mM HEPES 3 mM ZnCl_2_ pH 5.3, and buffer 2: 10 mM HEPES 3 mM NiCl_2_ pH 7.0 (Sigma-Aldrich Chemical, Poole, Dorset, UK). Oscillation of the cantilevers at their fundamental resonant frequency was driven using ‘blueDrive’ photo-thermal excitation. Photothermal excitation is a new technology developed by Asylum research that provides a more stable and controlled form of cantilever oscillation. This is achieved by positioning a laser beam with wavelength 425 nm and modulated power directly onto the cantilever’s bimetallic strip, as opposed to the traditional piezo-acoustic method, which mechanically oscillates the tip holder causing more potential disturbance of the samples. Localised heating by the blue laser causes the cantilever to bend due to the bimetallic strip effect and modulation of the power causes the probe to oscillate at an accurately controlled frequency and amplitude. The laser power modulation frequency was set at the fundamental resonant frequency of the cantilever (1.37 MHz) and the power level 124.8 μW set to generate an appropriately small oscillation amplitude (∼1 nm). The feedback loop control set-point was also kept at a very low level of damping of the cantilever’s free oscillation (∼5–10%) to minimise the loading force on the molecules. The AFM tips used were Arrow UHF-AuD (NanoWorld AG, Neuchâtel, Switzerland). Scan rates were set at 1.5 Hz.

### 2.2 Preparation of xanthan solutions

The xanthan used in this study was a powdered food grade xanthan gum (Keltrol RD, CP Kelco, Atlanta, GA, USA). ‘RD’ stands for a readily dissolvable product. The stock solution was prepared at a concentration of 1 mg ml^−1^ in pure water (18.2 MΩ). The xanthan powder was dispersed immediately after addition to the water at 22 °C by stirring. It was then left for 30 min to hydrate before heating to 95 °C for 60 min to completely disperse the molecules. The stock solution was allowed to cool to room temperature (22 °C) and then diluted to 3 μg ml^−1^ into either water (method 1, below), or the aqueous buffers (method 2, below). The diluted solutions were then re-heated to 95 °C for 60 min to reduce any aggregation and allowed to cool back to 22 °C prior to the AFM imaging preparation procedures.

The additional heating step was merely to ensure that the same structure and proportions of soluble/aggregate fractions are present in the test material (renatured state). Gulrez et al. (2012) investigated the effect of heat treatment on xanthan aqueous solutions (4 mg/mL) dissolved in distilled water, which was subsequently diluted to contain 0.1 M LiNO3 prior to injection into the GPC-MALLS system. They demonstrated that heating xanthan aqueous solution up to 40 min at 85 °C resulted in similar molecular weight parameters (i.e. weight average molecular weight, % mass recovery and polydispersity). Further heating up to 60 min resulted in an increase in the mass recovery and a slight increase in the molecular weight as a result of disassociation of large aggregates initially retained on the 0.45 μm filter. The molecular weight is reduced to almost half with full mass recovery and an increase in the polydispersity (from 1.64 to 2.75) when the diluted solution was heated for 2 at 85 °C.

### 2.1 AFM imaging preparation procedures

Two methods were used to physisorb xanthan molecules onto freshly-cleaved muscovite mica (Agar Scientific, Cambridge, UK).

#### 2.2.1 Method 1: drop deposition (includes drying)

A 3.5 μl droplet of xanthan at a concentration of 3 μg ml^−1^ in water was placed onto the freshly-cleaved mica and left to evaporate at room temperature (22 °C). When fully dry the sample was then placed into the liquid cell of the AFM and imaged in the aqueous buffers described in Section 2.1.

#### 2.2.2 Method 2: in-situ adsorption (no-drying)

100 μl of the buffer-diluted xanthan solution (3 μg ml^−1^) was placed directly into the liquid cell of the AFM, which contained freshly-cleaved mica and then imaged as described in Section 2.1.

## 3 Results

**Fig. 1:**
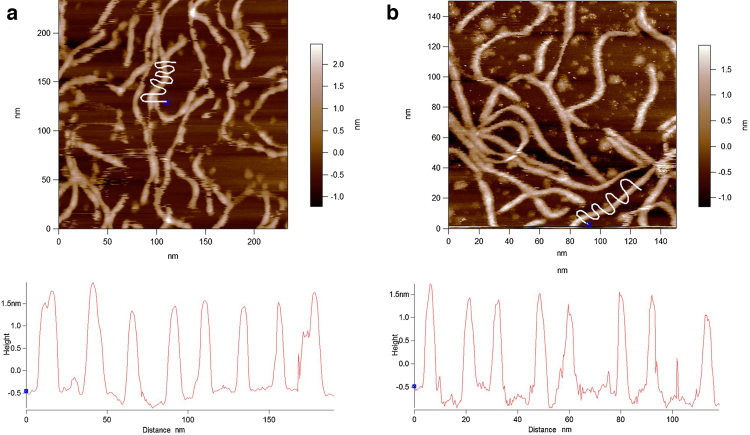
Early stage images of xanthan on mica in aqueous buffer. (a) Method 1, drop-deposited, imaged in buffer 1. (b) Method 2, *in-situ* adsorbed from and imaged in buffer 2. Bottom panels: Line profiles depict the heights of the features beneath the white lines in the images.

Fig. 1 displays an example of the data that were always obtained at the early stages of imaging xanthan in aqueous buffers, prepared by both methods (Fig. 1a drop deposition, Fig. 1b *in-situ* adsorption). The swirly white lines over one molecule in each image illustrates the location of the cross-sections that are profiled in the graph beneath the images. The reason they are swirly lines is to enable repetitive quantification of the height of the molecules. The average value in both cases is 2.06 ± 0.19 nm. Measurement of height is the most accurate way to quantify the diameter of polymers with AFM, as lateral dimensions are significantly oversized by probe-broadening (Morris, Kirby, & Gunning, 2009). The value of the height of this chain is slightly larger than the predicted width (1.8 nm) of xanthan in the double helical form (Millane, 1990). The linearity of the chains demonstrates rigidity. This provides evidence that at this early stage, despite there being no secondary structure visible within the chains, xanthan is not in a disordered conformation. Previous research has shown that xanthan in a disordered ‘random coil’ state has a significantly lower chain height than in the ordered state and also appears less linear due to its lack of rigidity (Gulrez et al., 2012). However, as mentioned above the interesting issue is that, despite the fact that the dimensions and linearity of the chains at this early stage are closer to the ordered conformation of xanthan, no periodicity was visible along any of the chains.

**Fig. 2:**
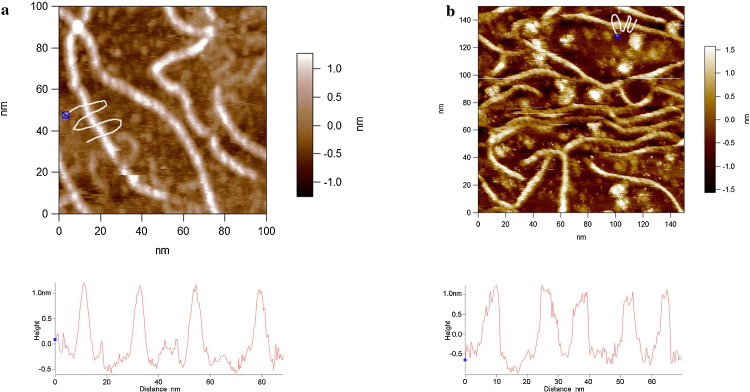
Resolution of the helical pitch of xanthan: (a) Method 1, drop-deposited, imaged in buffer 1. (b) Method 2, *in-situ* adsorbed from and imaged in buffer 2. Bottom panels: Line profiles depict the heights of the features beneath the white lines in the images.

After a certain length of time in the liquid cell of the AFM the helices were visualised in each of the samples prepared by the two different methods (Fig. 2a and b). Once the helices have annealed within their ordered state the width of the chains (accurately measured as height by AFM) is 1.6 ± 0.16 nm, which is more compact by approximately 0.4 nm than those measured prior to annealing. This height value is reasonably consistent with the width of xanthan obtained from x-ray fibre diffraction measurements (1.6–1.8 nm) (Millane, 1990). In buffer 1 the re-conformation of the helix took ∼16 hours and in buffer 2 it took ∼ 4 hours. The difference between the buffers containing Ni^2+^ and Zn^2+^ is the pH (7.0 & 5.3 respectively).

**Fig. 3:**
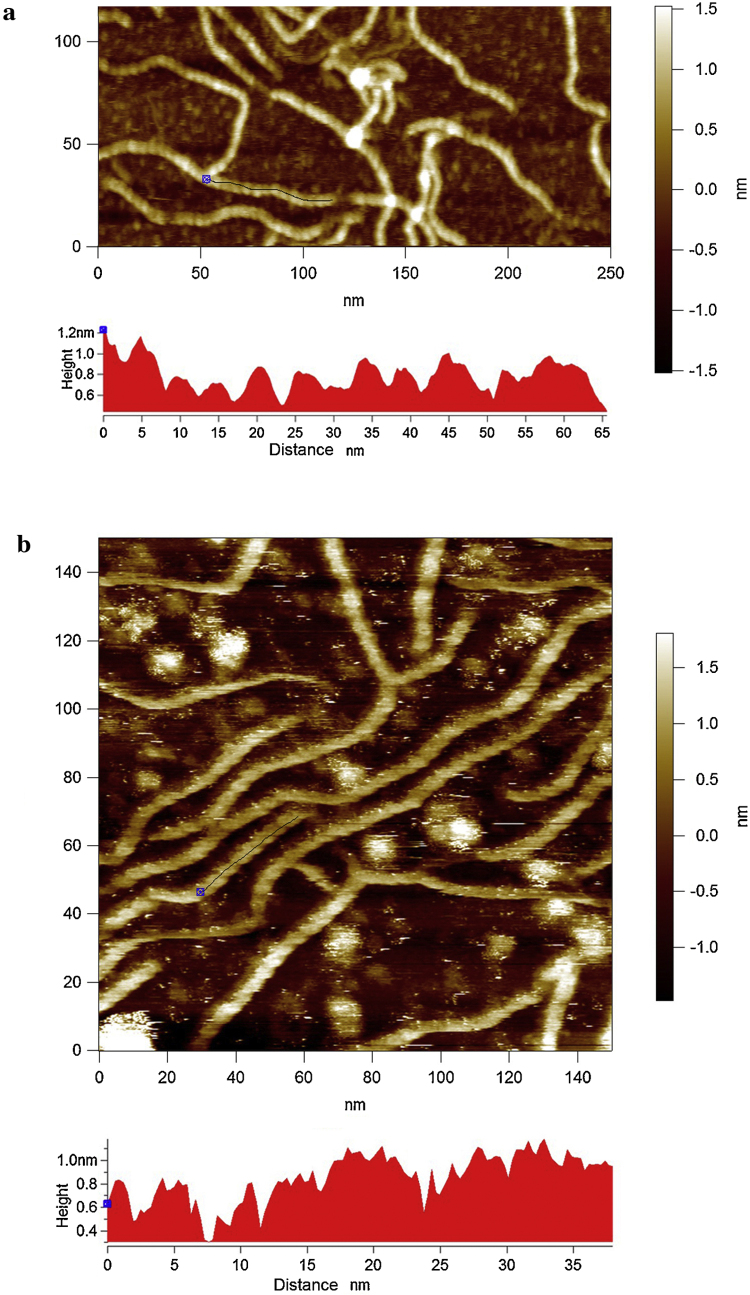
Helical pitch of xanthan (a) Method 1, drop-deposited, in the presence of Zn^2+^, (b) Method 2, *in-situ* adsorbed in the presence of Ni^2+^. Bottom panels: Line profiles depict the heights of the features beneath the black lines in the images.

**Fig. 4:**
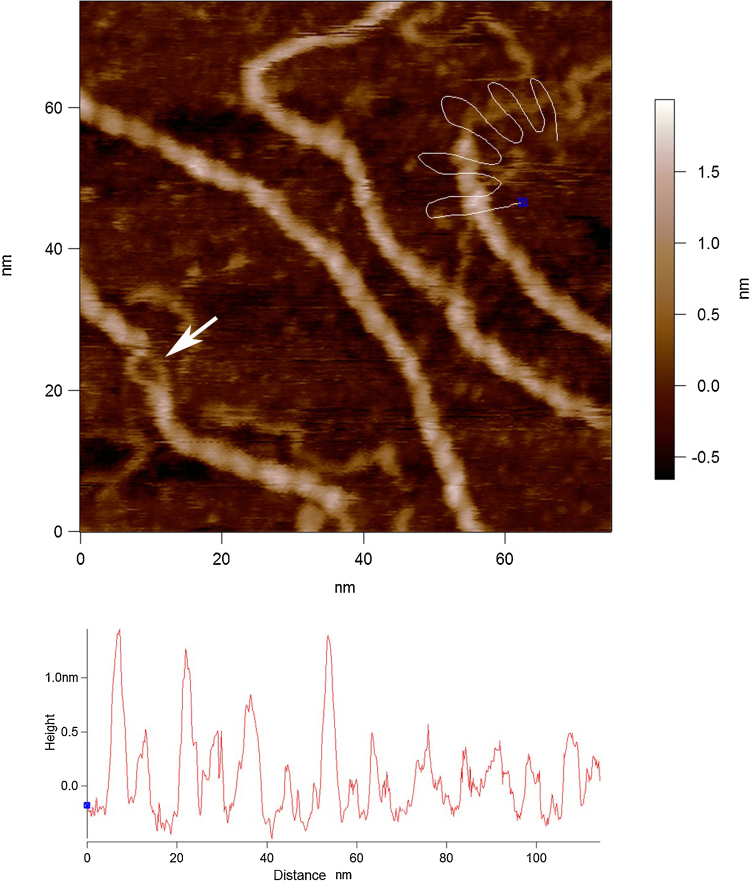
Method 1, drop deposited xanthan sample imaged in buffer 1 showing unravelling of the helices. Bottom panel: Line profiles depict the heights of the features beneath the thin white line in the image.

**Fig. 5:**
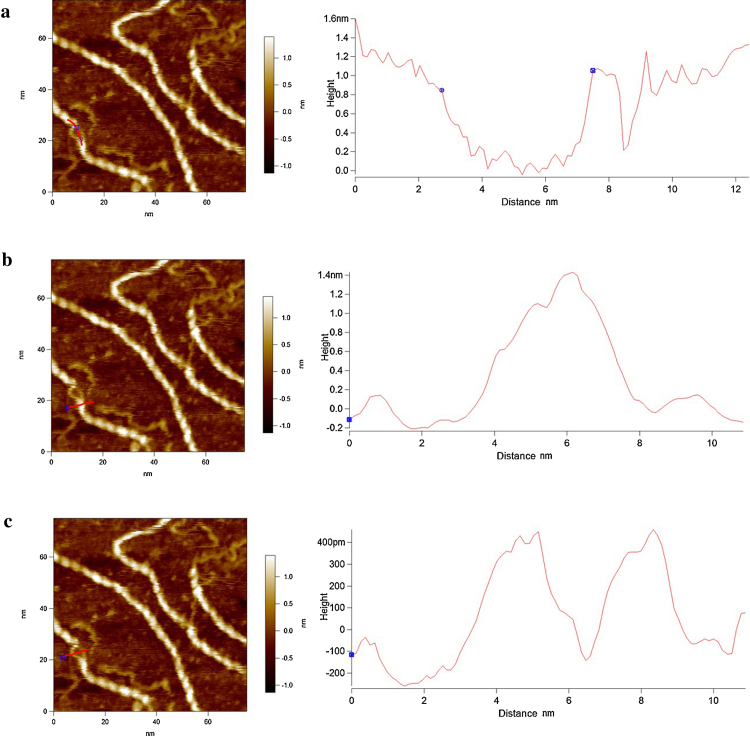
Additional measurements of the xanthan molecule containing the partially unravelled section. Line profiles (right panels) depict the heights and distances of the features beneath the red lines in the AFM images (left panels). (a) Profile of the gap created by the unravelled section, with blue markers (in both panels) labelling the transition zones. (b) Profile across the helical section. (c) Profile across the unravelled section. (For interpretation of the references to colour in this figure legend, the reader is referred to the web version of this article.).

The values of the periodicity observed after annealing have been quantified by line profiling along the molecules (Fig. 3a and b). This relates to the pitch of the helices, and the values obtained are 4.67 ± 0.29 nm in buffer 1 (Zn^2+^) and, although in Fig. 3b the line profile is noisier, the actual number of visible helical turns gives a similar value; 4.75 ± 0.11 nm in buffer 2 (Ni^2+^). These values are fully consistent with the previous x-ray fibre diffraction data (Millane and Narasaiah, 1990, Okuyama et al., 1980). There is a significant difference in terms of the consistency of the structural order between the different adsorption methodologies used. In the drop deposited samples (method 1) many of the chains display ends where the helices are unravelled (Figs. 2 a, 4, and 5 a–c) and the occasional case with a small, unravelled section of the helix arrowed in Fig. 4. The heights of the helical section and the unravelled section are quantified by the line profile in Fig. 4. The helical section has a height of 1.6 nm and the unravelled section has a height of 0.6 nm, which is less than half the value. The difference in the heights confirms that the taller section is not a simple dimerization of two single chains. It is therefore a more complex structural arrangement as expected for a helix.

Further analysis of this image with the unravelled section of the ordered structure is displayed in Fig. 5. The length of the unravelled section is 4.6 nm (Fig. 5a). The line profiles in Fig. 5b & creveal that the difference in the heights of each section are consistent with those measured on the other xanthan molecule in Fig. 4: helical section 1.6 nm (Fig. 5b) and unravelled ‘mid-section’ 0.6 nm (Fig. 5c). The height equivalence and elimination of periodicity confirms full disordering of the unravelled ‘mid-section’. The fact that the length of the unravelled section is no longer than the helical pitch observed in both buffers (Fig. 3) indicates that it is unravelling of the helix into two disordered strands.

**Fig. 6:**
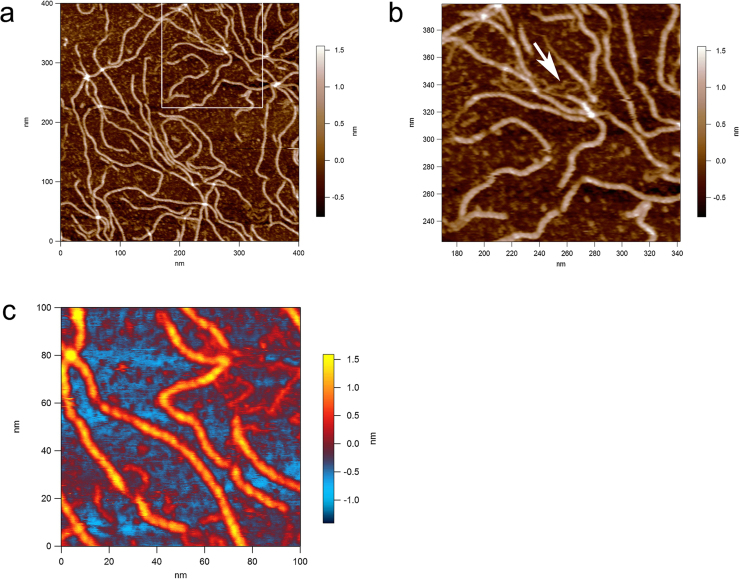
(a) Hairpin loop images: Helical xanthan molecules with unravelled ends. (b) white box marked region electronically zoomed, and (c) second hair-pinned molecule.

The images in Fig. 6 reveals further evidence for a double helical conformation of xanthan; one of the proposed helical structures suggested to be formed in a dilute solution. The suggestion is that xanthan’s double helix dissociates into two single chains at a denaturing concentration (≤1 mg ml^−1^) but, despite it seeming inconsistent, there is a possibility that single chains can reconstruct the intramolecular double helical structure in an anti-parallel manner with a hairpin loop at one end during the renaturation process (Matsuda et al., 2009). The arrow in Fig. 6b shows that occasional predicted hairpin loops do indeed exist and a second one is shown in Fig. 6c (an alternatively coloured version of Fig. 2a).

**Fig. 7:**
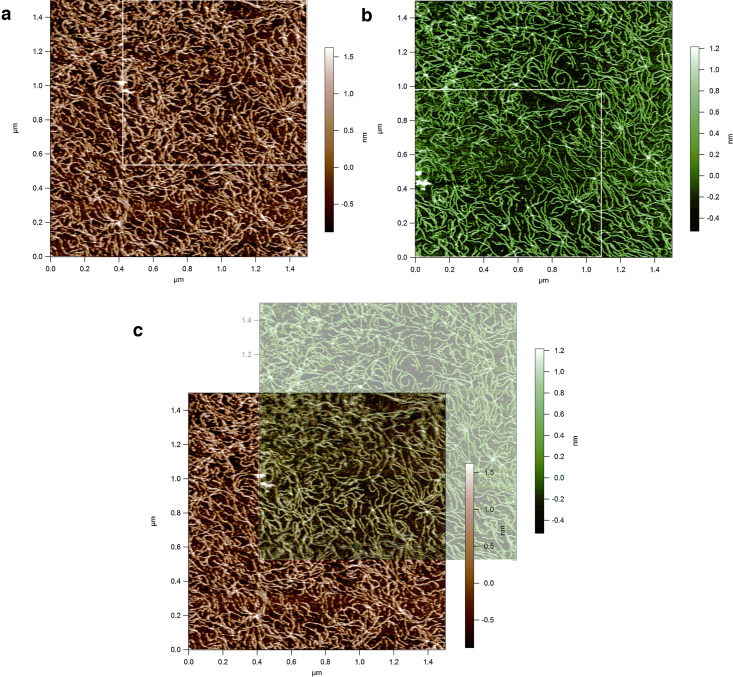
(a) Example images of the stability of the xanthan molecules over a 16 hour time period in buffer 1. (a) at 1 hour, (b) at 16 hours, and (c) images overlaid. (For interpretation of the references to colour in the text, the reader is referred to the web version of this article.)

Although the conformation of the xanthan changed with time to the fully ordered helical state, Fig. 7 shows a set of images demonstrating that the molecules remained stably attached to the mica in virtually the same positions over the longest investigated time period of 16 hours in the liquid cell in buffer 1. Fig. 7a was the last image taken during the initial stages when no helices were observable and Fig. 7b was the first image taken the following day when the helices were observable. Despite there being a small amount of drift between Fig. 7a and b (∼500 nm to the top right) the white box marker shows the matching regions in both images with no significant movement of the molecules themselves. Fig. 7c shows how consistent the position of the molecules is in both scans by overlapping the images. The later image (Fig. 7b) has been placed on top of the earlier image (Fig. 7a) and set to a different colour (green) with an opacity of 42% so that both can be visualised.

## 4 Discussion

In solution, screening of the xanthan’s charged groups by counterions can be achieved rapidly at an optimal concentration of salt because they are mobile and can sustain an equilibrium state. Previous research has shown how sensitive the helical conformation of xanthan is to the level of salt in the solution (Bejenariu et al., 2010, Brunchi et al., 2014, Matsuda et al., 2009). The two aqueous buffer solutions used for imaging the xanthan in the present study included divalent counterions (Ni^2+^ and Zn^2+^) at optimal screening concentrations for two purposes: The principal one is to facilitate adsorption of xanthan onto the mica so that it can be imaged in liquid. Without sufficient screening of the electrostatic repulsion between negatively charged water soluble molecules and mica there is no possibility of successfully imaging molecules in aqueous liquids because they will desorb from the mica surface, even if they have been previously deposited by air drying. The two counterions (Ni^2+^, Zn^2+^) were discovered to be optimal for binding DNA to mica in aqueous buffers due to their small ionic radii (0.69 & 0.74 Å respectively), which allows them to fit into the cavities above the recessed hydroxyl groups in the mica lattice (Hansma & Laney, 1996). In addition, Ni^2+^and Zn^2+^ have anomalously high enthalpies of hydration which is proposed to enable them to form strong complexes with ligands other than water (Hansma & Laney, 1996). The second aspect to be considered in the present study is that divalent counterions cause stabilisation of xanthan’s helical conformation in solution at concentrations ≥1 mM (Brunchi et al., 2014, Bejenariu et al., 2010). This is crucial because the xanthan had to be diluted to extremely low concentrations (≤3 μg/ml^−1^) to ensure that sub-monolayer adsorption was achieved on the mica surface. At higher concentrations the mica surface becomes overcrowded with multilayers of xanthan, which prevents resolving the individual molecules.

When the xanthan molecules adsorb to the mica, which has a heterogeneous surface charge distribution, the situation is different than in solution. The localised electrostatic interactions that occur between the charged groups present on the polysaccharide chain and the solid mica surface are highly unlikely to be optimal, since on the mica the spatial distribution of the charged groups are fixed so they cannot move and adapt to the charged groups on the xanthan molecule. Theoretical modelling studies demonstrated that heterogeneously charged polymers adsorbing to heterogeneously charged surfaces do so by adopting their shape, interpreted as pattern recognition (Chakraborty & Bratko 1998; Golumbfskie, Pande, & Chakroborty, 1999). It has been previously reported that alteration of xanthan solution pH affects the transition temperature (Bejenariu et al., 2010) although the pH differences investigated in that study were significantly larger (3, 7 & 13) than in this study. Another difference between buffers 1&2 is the water substitution rates of Nickel compared to Zinc (Ni^2+^ 2.7 × 10^4^/s, Zn^2+^ 5 × 10^8^/s; Kobayashi, Nagayama, & Busujima, 1998). This is therefore more likely to be the reason that the re-annealing time of the xanthan helices is significantly different in the buffers used in the present study.

This clearly has an effect on xanthan’s ultrastructure, but there are two potential reasons. The initial and more obvious interpretation of the time requirement to visualise the helical periodicity due to the slight streakiness seen in the early stage images (specifically Fig. 1a) is that the xanthan may not be sufficiently stably attached to the mica for high-resolution imaging at the early stages. However, there is relatively strong evidence that loose binding may not be the only reason. The images in Fig. 7 show that nearly all of the molecules remained in precisely the same position on the mica over the entire experiment time so they must have been reasonably bound from the very start. This is probably due to the combination of the ‘pattern recognition’ shape adoption of the molecules binding to the most suitably charged regions of the mica and also the strength of the stiff polymer network. This provides clear evidence that the molecules that adopted their conformation from partially disordered to the fully ordered helical state over the time period were those which were imaged during the initial stages, and not a set of other xanthan molecules on the mica.

Based upon this a unique interpretation of the lack of observable periodicity in the early stage images suggests that, even if it is in a fully ordered conformation in solution, xanthan’s helical order is probably distorted as it initially adsorbs to the mica. The distortion is however limited; the height measurements and linearity illustrated in Fig. 1 demonstrate that the distortion is not a full helix to coil transition but clearly is sufficient to remove any observable periodicity along the polymer chain. This enables interpretation of the height reduction of the chains from 2 nm at the initial stage (Fig. 1) to 1.6 nm (Figs. 2, 4, and 5 b). Helical formation was therefore likely due to an annealing compaction of the slightly distorted structure.

There is a fortunate benefit from mica’s distorting influence; certain sections of the chains (Fig. 2, Fig. 4) do not fully re-order which enables visualisation of the composition of the helix. It is clearly double stranded. The fact that the length of the unravelled section in Fig. 5a is no longer than the helical pitch observed in both buffers (Fig. 3) indicates that it is unravelling of the helix into two disordered ‘strands’. Although this seems obvious it provides additional information on interpreting the nature of the helical structure, single or double helical? If it was a single helix it would not produce two strands. Unravelling of a double helix would produce two fully disordered strands.

The visualisation of the hairpins (Fig. 6) provides significant assistance in the interpretation of the partially unravelled middle section of the other xanthan chain in Fig. 4, Fig. 5. In summary, this combination of images provides further direct evidence that xanthan’s ordered structure is a double helix. In addition, for a double helix to be formed by a single chain it will wrap around itself in anti-parallel conformation. The images obtained in this study demonstrate that xanthan can, by intra-molecular association, form an anti-parallel double helix. For the majority of images which do not show hair-pin loops the similar height and pitch suggest these are either parallel or anti-parallel double helices formed by inter-molecular association of two xanthan chains.

The alternative that these molecular structures could be formed by association of two single helices is unlikely for the following reasons. The structures containing hairpin loops are the same as those that do not show such loops. In studies of the related xanthan-like polysaccharide acetan (Kirby, Gunning, Morris, & Ridout, 1995) it was possible to image, by AFM, side-by-side association of helices in an aligned liquid crystalline monolayer showing the expected pitch and height for the helices. This shows that AFM would distinguish between a double helix and paired single helices, since both single helices in the pair would need to bind to the mica. Further, as discussed later, although there is a stereo-chemical basis for dimerization of chains to form a double helix, there is no stereo-chemical basis for dimerization of single helices, which would restrict aggregation to dimers, or explain why it extends along the complete length of the molecules.

In method 2, the samples prepared by *in-situ* adsorption of the xanthan from the buffers containing the divalent counterions, unravelling of the helices was not detected (Figs. 2 b, and 3 b). This reflects the state that the xanthan is likely to be in prior to its attachment to mica in the different methods. In method 1 (drop deposition from pure water) it is likely to be in a disordered conformation at the initial stage, due to the very low concentration of the xanthan, which also means that the solution is very low in ionic strength. As the water droplet evaporates the polysaccharide concentration and ionic strength increases driving xanthan towards its helical conformation, but not surprisingly there can be many sections of the chains that do not have time or the correct conditions to fully re-order. In method 2 the molecules will predominantly be in the helical conformation in the solution due to the optimal concentration of the divalent counterions in the buffer, and also the heating/cooling steps in the sample preparation procedure (Gulrez et al., 2012) before they adsorb to the mica. As can be seen in the examples images in Figs. 2 b, and 3 b this greatly reduces the probability of any fully unravelled sections of their conformation.

If the secondary structure of xanthan was one of the other previous interpretations (Norton et al., 1984) based upon the measurements of the kinetics of conformational ordering, namely dimerization of single helices, then two strands would also be a predictable outcome. Light scattering and optical rotation values came from point-by point equilibrium measurements, so this was not a kinetic “time lag” effect but a difference in the temperatures needed to trigger the onset of conformational ordering and the increase in molecular weight. Similarly, when the variable was not temperature but concentration of cadoxen, reduction in molecular weight began at substantially lower cadoxen concentration than loss of conformational order. However, that could not be attributed to slow kinetics of ordering, because the starting point was the ordered solid. That was interpreted as xanthan initially forming single helices in the disorder-order transition and then dimerising, but there is visual evidence in this study, which reveals that xanthan is not likely to be a dimerised single helix. A more recent study showed that when xanthan solution is treated by high pressure homogenisation there is a significant decrease in the molecular weight but the measurements of molecular weight per unit contour length of the rod (M_L_) suggests it is still double helical, and even after storage of the solution for 3 days the molecular weight parameters hardly changed (Gulrez et al., 2012).

Dimerization would be resolvable along the entire ‘fibre’ if the single helices were parallel-aligned. The only potential reason that dimerised single helices could be visually concealed until they become disordered would be if they wrap very compactly around each other and hence appear as single rather than double fibres. But in that case the heights of both the ordered sections and the separated ‘strands’ would not match the values quantified in Fig. 4, Fig. 5. The ‘strands’ would not drop to such an extent because the modelled width of xanthan as a single helix was the same value (1.6 nm) as that of the double helix (Norton et al., 1984) and of course that means the ordered section if it was composed of two wrapped single helices would very likely be taller than 1.6 nm.

Another hypothesis is that for dimerised single helices to separate and disorder to reach the low height of the ‘strands’ in all the unravelled sections visible in the AFM images then the length of the disordered gap in Fig. 5a would be different than the measure of the pitch of the ordered section if it was composed of two single helices that ravel around each other. Therefore, the length of this unravelled ‘mid-section’ potentially provides further direct visual evidence favouring that the secondary structure of xanthan in its ordered state is a double helix.

Although this study is based solely upon AFM image data the conclusion is not just from the visual evidence. The combination of images with topographical quantification has enabled the interpretation of the AFM data in relation to the previous predictions of xanthan’s secondary structure from all of the other techniques, which have been carried out over many years. Therefore, the height data from all of the ordered and disordered sections visualised in the AFM images provides confirmation that the xanthan observed in this study is double helical. Note that these facts are based on the AFM-observed structures which are of course limited to those that bind to mica and no dimerised single helical versions were detected in any of the images captured (n = 41, typical molecules per image = 50–250), but there may still be a possibility that in solution stochastic variations in xanthan’s confirmation can exist due to molecular mobility. It is therefore possible that the controversy over single or double helix formation may have arisen due to experiments done under different experimental conditions which favoured intra- or inter-molecule association: double helices can form by intra-molecular association of single chains.

It is interesting to consider stereo-chemical reasons why the helical structure is composed of two interacting chains. The nature of the primary structure of xanthan suggests that the distribution of sidechains along the backbone results in an uncharged and a charged face of the cellulosic backbone. Association of the uncharged faces of the backbone, and a twist into a 5-fold helix to optimise the distribution of charge on the helix, could explain the formation of the double helix. This would be consistent with intra-molecular association (anti-parallel) or inter-molecular (anti-parallel or parallel) association. Such a model would be consistent with the observed 6-fold helical complex with a pitch of 5.6 ± 0.1 nm, formed between the xanthan-like polysaccharide acetan and the glucomannan konjac mannan (Ridout, Cairns, Brownsey, & Morris, 1998) and the proposed double helical structure, which contains both a konjac mannan (uncharged) and a single acetan chain (charged) within the helix (Chandrasekaran, Janaswamy, & Morris, 2003). Acetan like xanthan forms a 5-fold helix with a pitch of 4.8 nm (Morris, Brownsey, Cairns, Chilvers, & Miles, 1989). The transition to a 6-fold helix could result from the different optimised distribution of charge along the helical complex.

## 5 Conclusions

The ability of AFM to resolve polysaccharide molecules at sub-molecular resolution and the distorting effect of the heterogeneously charged substrate, mica, has provided the first ever direct visual evidence which confirms that the proposed anti-parallel double helical ultrastructure of xanthan can be formed through intra-molecular association. For the majority of ordered structures, which do not show the presence of loops, both anti-parallel and parallel models for the double helix formed by inter-molecular association are possible. The data in this study confirms that the precision of the formation of xanthan’s secondary helical structure is indeed sensitively driven by an optimisation of intra-molecular charge screening. The AFM data suggests that xanthan’s predominant equilibrium structural conformation is a double helix.
